# Food for thought: food availability alters crayfish perception of fear and safety in risky habitats

**DOI:** 10.1093/cz/zoaf076

**Published:** 2025-12-03

**Authors:** Madison J Alexander, Paul A Moore

**Affiliations:** Laboratory for Sensory Ecology, Department of Biological Sciences, Bowling Green State University, 217 Life Sciences Building, Bowling Green, OH 43403, USA; University of Michigan Biological Station, Department of Ecology and Evolutionary Biology, 9133 Biological Road, Pellston, MI 49769, USA; Laboratory for Sensory Ecology, Department of Biological Sciences, Bowling Green State University, 217 Life Sciences Building, Bowling Green, OH 43403, USA; University of Michigan Biological Station, Department of Ecology and Evolutionary Biology, 9133 Biological Road, Pellston, MI 49769, USA; J.P. Scott Center for Neuroscience, Mind, and Behavior, Department of Pyschology, Bowling Green State University, 300 Pyschology Building, Bowling Green, OH 43403, USA

**Keywords:** aquatic ecology, behavioral ecology, landscape of fear, predator–prey, refuge quality, sensory ecology

## Abstract

Recent evidence shows that prey behavior is mediated by information regarding predators, refuges, and possibly food. However, the ways in which these 3 factors influence prey behavior in tandem with one another is still largely unexplored. We exposed rusty crayfish (*Faxonius rusticus*) to largemouth bass (*Micropterus nigricans*) odors and quantified sheltering and foraging behaviors. We presented crayfish with shelters of varying quality in 2 spatial arrangements and changed the location of a food source, *Chara* spp., to be near each shelter in different treatments. In this way, we created both a gradient of fear and of safety independently from one another while attempting to alter the perception of these gradients with changes to food location. We found that predator odor, refuge spatial arrangement, and food location were all important for crayfish behaviors. First, we found that time sheltering and time near shelters were both significantly impacted by interactions of shelter quality and arrangement. Crayfish only spent more time in and near upstream high-quality shelters when *Chara* spp. was also upstream. Next, we found that the average gape ratio of bass used in trials significantly altered the total amount of *Chara* spp. consumed, time spent foraging, and crayfish movement. The results of this study show the importance of information related to risk, safety, and food in altering prey behaviors, and these factors are used in combination. Specifically, the presence of a food source and the proximity of a food source to a shelter may affect where and when a prey organism shelters.

## Graphical Abstract

**Graphical Abstract:**
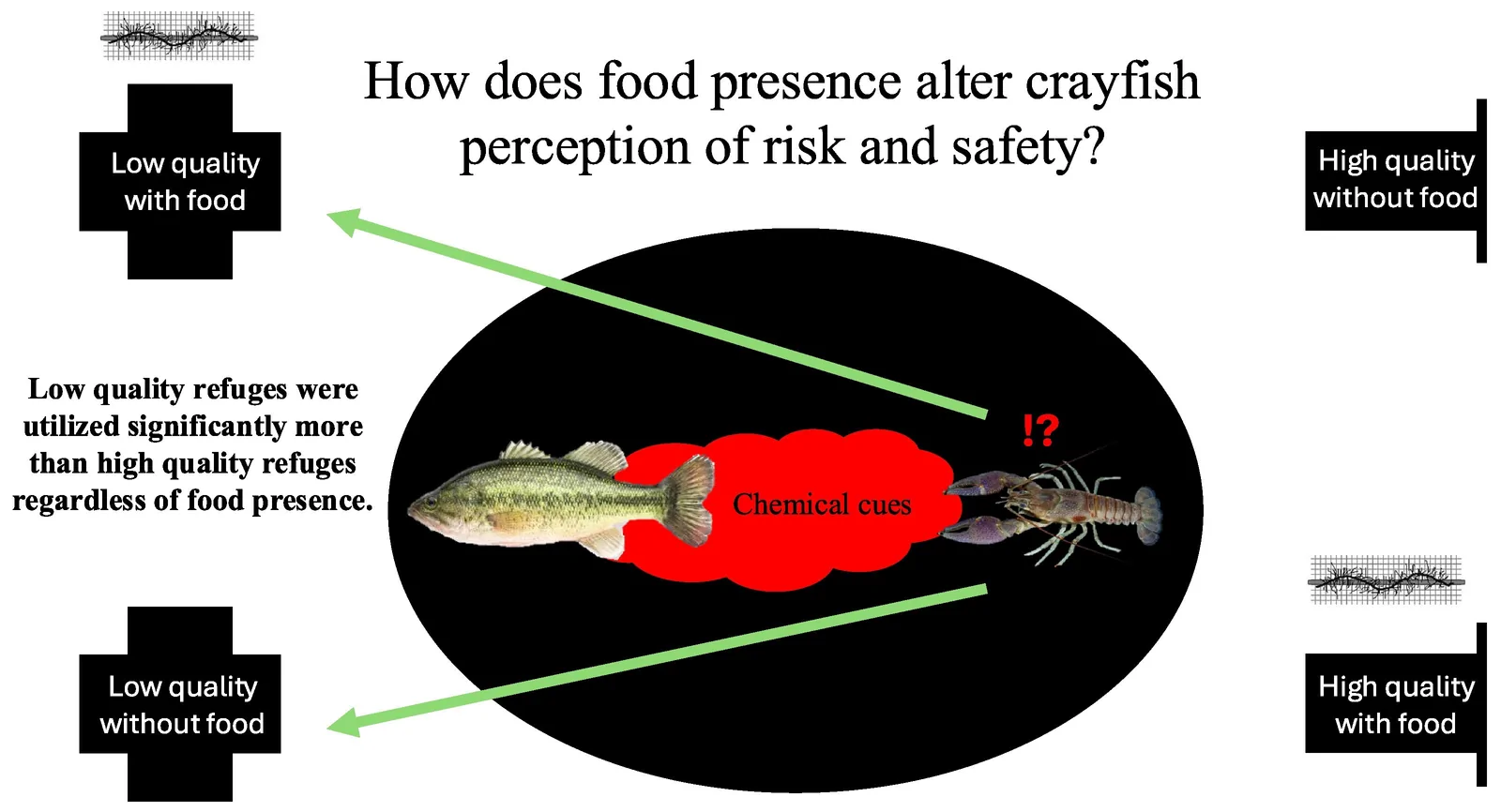

One of the strongest factors in structuring prey populations and how the individuals in these populations behave is predation (Brown et al. 1999; Ripple and Beschta 2004; Gaynor et al. 2019). Within this predation framework the structuring factor is fear of predators as well as safety from refuges within a habitat. Prey species have been known to shift reproductive times, where foraging takes place, and even the time of day individuals are active in response to predator presence (Preisser et al. 2005; Laundré et al. 2010). Similarly, safety within a habitat in the form of refuges has also been shown to alter prey behavior in high-risk scenarios (Luttbeg et al. 2020; Kelleher et al. 2021). The availability of varying quality refuges as well as the spatial arrangement of refuges within a habitat alters the time prey spend sheltering at different risk levels (MacKay et al. 2021). Taken together, prey must utilize information based on both fear and safety in order to make decisions under threat and decrease the chance of predator capture. Models show that prey are measuring risk and reward from the information gathered about predation threat and refuge location and quality. However, less described within the literature of predator–prey ecology is what can change the perception of risk and reward and, thus, the behaviors that follow.

Food availability within a habitat is an important driver of where and when organisms are active (Gonzalez-Ortiz et al. 2014). When considered in light of predation, ecologists often measure how time spent foraging, food preference, and even the total amount of food consumed changes (Laundré et al. 2001; Charalabidis et al. 2017). Foraging or consumption are often used as measures of fear an organism experiences, such as in classical optimal foraging theory or in experiments centered around giving up densities (Kotler and Blaustein 1995). However, we often do not consider that, in some situations, the presence of a food patch and the patch's proximity to a refuge may be what is informing prey behavior when risk is high. Certainly, various models and experiments have been conducted that examine the relationship between food patches’ proximity to refuges and/or predators, but these results tend to focus on ensuing behavioral changes that exert some force at the organismal or population level (Holbrook and Schmitt 1988; Brown 1999; Crowell et al. 2016). An area less examined is seeking to understand when food availability may increase or decrease the desirability of different types of refuges in varying levels of risk. Prey perception of relative risk and safety levels is one of the first steps in the decision-making paradigm that ultimately leads to an array of behaviors that prey may perform. Thus, understanding how prey organisms titrate fear and safety can only be done if we understand the components organisms use to calculate these factors.

In order to evade predation, prey organisms must be able to measure risk within the environment. This measurement is accomplished by obtaining various pieces of information (e.g., predator abundance, relative size ratios, type of predator, etc.) from the sensory landscape, which is the sum total of all stimuli present in an organism's environment (Weissburg et al. 2014; Wagner and Moore 2022). For instance, visual stimuli can show relative distances that predators are from prey. Similarly, chemical cues can provide predator dietary preferences and even satiation level to prey (Smith and Belk 2001). Each of these cues are a factor that prey may utilize in order to calculate the relative risk a predator poses. However, less risk is not necessarily equivalent to safety. For instance, running away from a predator, but not toward a refuge, may momentarily relieve predation pressure, but if no refuge is present, risk may have decreased whereas safety has not actually increased. Similar to risk calculation, prey may utilize certain factors such as nearness to a refuge, whether a refuge is occupied, or physical attributes like number of openings and size of a refuge to calculate the relative safety offered (Caley and St. John 1996; Kramer and Bonenfant 1997; Scharf et al. 2006). Because not all refuges provide the same level of safety, prey organisms must utilize information about both the refuge and the level of risk to make sheltering decisions. Finally, factors such as the size of a food patch, the location of a patch, and the type of food present in a patch are all pieces of information that organisms use to decide when, where, and how long to forage (de Knegt et al. 2007; Morrison et al. 2010). Because foraging can be energetically costly and decreases vigilance, information regarding a specific food patch as well as the surrounding habitat is something prey organisms must consider. Though these are certainly important factors in calculating risk, safety, foraging behaviors, there is still a need to understand if prey organisms consider these factors singularly or if they are combined in calculating risk and safety. Previous work has shown that prey organisms utilize information related to both risk and safety in a habitat (Ducsai et al. 2023; Alexander and Moore 2025), but we still know little about what components, such as food resources, may go into altering the perception of these decision-making factors.

The aim of this study was to better understand how prey perception of fear and safety might be altered by the proximity of food resources, so we created a system in which we spatially modulated these 3 factors: fear, safety, and food. In many aquatic habitats, chemical cues typically mediate predator–prey interactions (Brönmark and Hansson 2000). Because of this, the focal organism for this study was the rusty crayfish (*Faxonius rusticus*), which is a known keystone species and ecosystem engineer (Creed and Reed 2004; Usio and Townsend 2004). Crayfish also utilize chemosensation to locate conspecifics, food, refuges, and even potential predators (Callaghan et al. 2012; Driscoll et al. 2020). Although crayfish act as a top predator for many types of benthic plants and macroinvertebrates, crayfish are also an important prey item for various fish species, so understanding the ways in which this species may alter their behavior is increasingly important (Reynolds et al. 2013). Previous work has also shown that crayfish choose refuges based on the total number of openings on the refuge itself (Martin and Moore 2008). Thus, there is some perceived quality to refuges. What remains unclear is whether or not this perceived quality may be altered by food presence and proximity to refuges. We exposed rusty crayfish to predator odors while simultaneously presenting crayfish with refuges of different quality and proximity to food resources and recorded sheltering and foraging behaviors. We hypothesized that crayfish would use high-quality shelters significantly more than low quality shelters when risk was high regardless of food location, and that low-quality shelters would only be utilized more when food resources were present and risk was low. We also hypothesized that overall movement of crayfish would decrease in the presence of predators.

## Materials and methods

### Experimental design

To address these aims, we created a 2 × 2 × 2 design to test how rusty crayfish shelter usage was impacted by a gradient of safety, a gradient of fear, and food availability in a risky habitat. Because previous work has shown that rusty crayfish shelter usage is altered by both fear and safety cues (Alexander and Moore 2025), we were interested in titrating the potential attractiveness of shelters by adding food resources to 1 of 2 shelters of varying quality in varying spatial arrangements and risk levels. The factors for this project were the spatial arrangement of shelters of varying quality (which creates a gradient of safety) in our system, the availability of food resources at one of these shelters, and the presence or absence of predators (risk). Thus, all trials without predator odor were considered control trials. In order to create a risky habitat, we exposed prey (crayfish) to fish predator chemical cues. The proximity of a refuge to predators has been shown to have significant effects on the usage and length of stay demonstrated by crayfish (Ducsai et al. 2023), which allowed us to create a fear gradient within experimental set-ups. All trials were video recorded to quantify crayfish behaviors, and food resources were weighed to calculate total food consumption in each trial. We had a total of 8 different treatments with a total N = 166 (Fig. 1; Table 1). All crayfish were used only once to avoid conditioning in trials. All video trials lasted for a total of 4 h.

**Figure 1:**
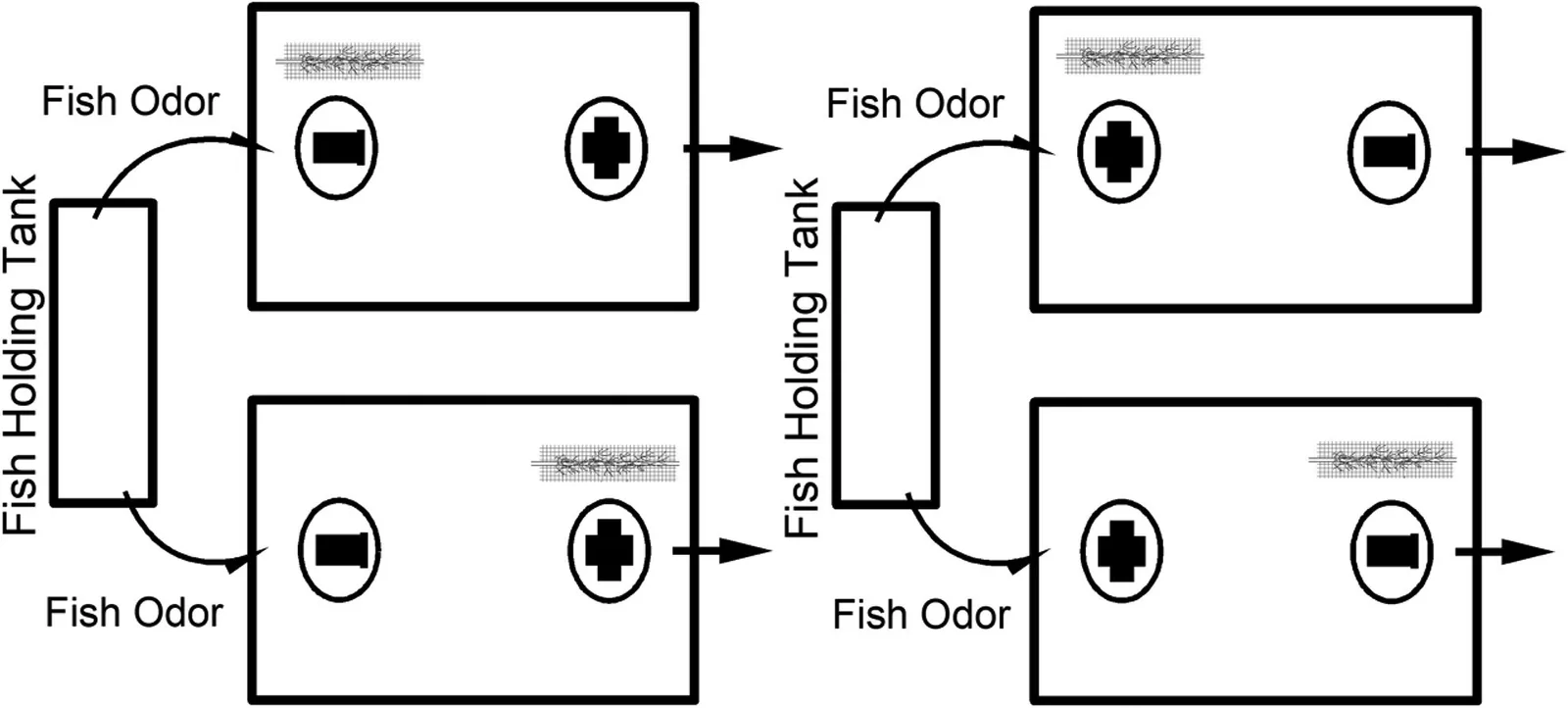
This figure depicts the mesocosm design of each of our shelter spatial arrangements and *Chara* spp. locations. Mesocosms on the left-hand side show a 1-4 arrangement, with 1-opening shelters closest to the source of odor introduction, whereas mesocosms on the right show a 4-1 arrangement. *Chara* spp. location is also depicted as being either near the 1-opening or 4-opning shelter in each mesocosm. *Chara* spp. location is either upstream (near the source of odor introduction) or downstream (near where water exits the system). Mesocosm dimensions were: 100.8 × 80.6 × 80.6; l × w × h cm.

**Table 1:** Summary of the treatments conducted where the shelter arrangement treatment lists shelter quality (high or low) proximity to predator cues.

Shelter arrangement	Predator	Food location	*N*
1-opening near cue	Present	Near 1-opening	20
1-opening near cue	Present	Near 4-opening	21
1-opening near cue	Absent	Near 1-opening	21
1-opening near cue	Absent	Near 4-opening	21
4-opening near cue	Present	Near 4-opening	21
4-opening near cue	Present	Near 1-opening	20
4-opening near cue	Absent	Near 4-opening	21
4-opening near cue	Absent	Near 1-opening	21

Predators are listed as present or absent (controls). *Chara spp.* location is shown as either being near 1- or 4-opening shelters. Finally, the total *N* for each treatment is shown.

### Organism collection and housing

#### Prey

One hundred sixty-six female form II (non-reproductive) rusty crayfish (carapace width = 1.9 ± 0.02 cm (mean ± SEM); range of values = 1.4–2.8 cm) were captured using hand nets from Maple Bay State Park on Burt Lake in Emmet County, MI, USA (45.4887°N, 84.7068°W). Crayfish captured were potentially previously exposed to bass in natural settings. However, over the course of this project, crayfish and bass were isolated from one another until trials began. Because carapace width was necessary for calculating gape ratio as opposed to carapace length, we only recorded crayfish carapace width. Crayfish appendages were fully intact, as this could alter chemosensation and movement during trials. Crayfish were housed in steel flow-through 950 L cattle troughs (200 × 60 × 60; l × w × h cm) which were fed unfiltered river water from the East Branch Maple River. Water was delivered via a PVC delivery pipe (ID = 7.6 cm) and exited the holding tank via a standpipe covered with screening that kept water depth at approximately 60 cm. Naturally occurring detritus was allowed to accumulate in the tank for crayfish to feed upon, and shelters made from clay pot halves were also provided for crayfish to utilize. All crayfish carapaces were marked with Wite-Out (BIC Wite-Out 2-in-1 Correction Fluid) to allow for better visibility when reviewing behavioral videos. Wite-Out has been shown to not affect crayfish behavior (Fero and Moore 2008; Jurcak and Moore 2018). After completion of trials, crayfish were not returned to the river due to the invasive status of rusty crayfish in Michigan and were frozen according to collection permit requirements. Crayfish were housed outside under natural temperatures and light:dark regimes from 28 June to 15 July 2025.

#### Predators

A total of one hundred thirty-seven largemouth bass (*Micropterus nigricans*) (total length = 24.8 ± 0.02 cm (mean ± SEM); range of values = 18.5–32.5 cm) were utilized for this project. One hundred twenty were purchased from Harrietta Hills Trout Farm (Harrietta, MI, USA), and 17 was purchased from Imlay City Fish Farm (Imlay City, MI, USA). Bass were kept in flow-through 1,330 L cattle troughs (238 × 86 × 60; l × w × h cm) which were fed unfiltered river water from the East Branch Maple River. Multiple cattle troughs were maintained and housed fish, and there were never more than 40 fish in a cattle trough at one time. Bass were fed a diet of commercial fish food (Sportsman's Choice Trophy Fish Feed) once a day. Additionally, each trough was covered with mesh screening to prevent bass escape and the introduction of macroinvertebrates into bass water. Similar to crayfish, bass troughs also had standpipes covered with screening that kept water depth at approximately 60 cm. Bass were also provided with shelters in the form of PVC pipes. After trials were completed, bass were euthanized following established animal care and use guidelines. All bass were housed outside under natural temperatures and light:dark regimes from 28 June to 15 July 2025.

### Plant collection and storage

Samples of American muskgrass (*Chara* spp.) were collected from North Fishtail Bay of Douglas Lake, in Cheboygan County, Michigan, USA (45.5618°N, 84.6762°W). A macrophyte sampling rake was cast into mats of submerged vegetation to collect the aquatic plants. The collected resource was held in half of a plastic barrel (59 × 59 × 44; l × w × h cm) that acted as a storage stream and was filled with water from the East Branch Maple River. The halved tank had an overflow hole drilled into the side that allowed for water to constantly flow through. The storage stream was placed in open sunlight to mimic a natural environment. A surplus of samples was maintained and replenished when wilting occurred.

### Experimental mesocosms

Twelve artificial flow-through streams (100.8 × 80.6 × 80.6; l × w × h cm) were framed with cinderblocks and lined with 6 mil plastic sheeting (0.15 cm thickness). Three 208-*l* plastic drums (1 each for a group of 4 streams) served as constant head tanks for water delivery from the East Branch Maple River into each mesocosm. Water entered head tanks through 7.6 cm PVC pipes covered in nylon stockings (0.1 mm openings) to prevent macroinvertebrates from entering the head tanks and mesocosms. Each drum fed water to 4 artificial streams via 2 garden hoses each (ID = 1.9 cm). Water flowed through mesocosms at a rate of 0.1 ± 0.05 L/s per garden hose. Each mesocosm also had a gravity drip system (described below) in place to add bass chemical cues to trials via a gravity system connected to a constant head tank. All artificial stream bottoms had a sand substrate layer (depth ∼ 3 cm) to mimic surrounding natural systems as well as to provide a dark background for behavioral assay video analysis. This construction technique has worked previously in ecotoxicology projects (Steele and Moore 2019).

Each mesocosm also had 2 shelters of varying quality placed in a straight line in the center of the arena and parallel to the sides of the mesocosm (further described below). Previous work has shown that crayfish prefer shelters with fewer entrances (less entry possibilities for predators; Martin and Moore 2008). Thus, we utilized shelters with 1 (highest quality) and 4 (lowest quality) entrances. We also defined the space directly on top of each shelter as the shelter area, so as to have a metric to measure crayfish time spent on shelters during exploratory bouts. The shelter treatment had 2 conditions. The first shelter condition had shelters increasing in quality away from the stimulus introduction (i.e., the 4-entrance shelter was closest to where the cues entered the water whereas the 1-entrance shelter was furthest away) and hence termed 4-1. The second condition had decreasing shelter quality away from the stimulus introduction (i.e., the 1-entrance shelter was closest to where cues entered the water and the 4-entrace shelter was furthest away) and termed 1-4. Water exited mesocosms via a screened opening (28 cm × 12 cm opening with 1 mm × 1 mm screening) from the downstream end. A wooden frame held a single infrared DVR camera (Zosi ZR08ZN10) 1.1 m above the surface of the water in each mesocosm to record crayfish nocturnal behaviors. All cameras had a frame rate of 30 frames/s, and a red lightbulb was placed above each stream to illuminate the arenas. All mesocosms were placed outside.

#### Landscape of fear: cue delivery

A constant head tank pumped water from a bass holding tank containing chemical cues from 2 bass into artificial streams. The cue delivery system was comprised of 2 parts: the reservoir (291 L) and the delivery tank (1.3 L). The delivery tank was constructed from PVC pipe with an end cap that had 15 cm of tubing (ID = 0.5 mm) attached to the bottom that was used to deliver chemical cues into streams. Each tube was situated ∼5 cm above the substrate layer (which was approximately halfway up the water column) to mimic the area in the water column where bass would likely hunt. A Hydor pump (model: Seltz l30; 700 L/h) transferred water from the reservoir to the delivery tank. An overflow pipe (7.62 cm × 7.62 cm × 5.08 cm PVC Y) allowed for excess water to be diverted from the delivery tank back into the reservoir. The reservoir and delivery tank remained covered in order to prevent potential contaminants from entering the water as well as to prevent bass escape. All pumps were fixed to a timer (Black and Decker Grounded Outlet Timer with Waterproof Outlet Timer for Lights) and set to turn on from 0000–04000, which was the total duration of each trial.

#### Landscape of safety: shelter quality and arrangement

The pattern in which shelters were arranged as well as the quality of each shelter was altered in trials to create a gradient of safety. Two shelters of varying quality were constructed and placed in each mesocosm. Because crayfish prefer shelters with fewer openings (Martin and Moore 2008; Chibucos et al. 2015), we assigned shelter quality based on the number of openings present. All shelters were constructed of PVC pipes (10 cm × 8.5 cm) cut in half longitudinally. Shelters with a single opening (called 1-opening shelters) were made from straight pipes, with one end sealed, and shelters with 4 openings (called 4-opening shelters) were made from PVC half-pipe crosses. Shelters were painted black, and each “arm” of the shelter was marked on top with Wite-Out to improve visibility during behavioral trials. The utilization of shelters in this project could be similar to logs, rocks, or various other types of debris found in heterogenous riverine landscapes that may vary in the number of openings, and thus, perceived quality, that crayfish may encounter in a natural setting.

### Experimental protocol

Trial cycles began each morning at 0900 with the selection and measurement of crayfish for trials and ended at 0800 the next morning when crayfish were removed from mesocosms. The only criteria for crayfish to be selected for trials were sex, reproductive status, and intactness of appendages, as all crayfish used were fully intact form II females. All trials included one crayfish, and each crayfish received a rectangular Wite-Out mark on the back of their carapace. The carapace width of each crayfish was measured to the nearest 0.1 mm in order to calculate gape ratio throughout trials. Gape ratios were calculated by dividing carapace width with total bass gape (cm). This measure is commonly used to describe the relative threat bass pose to prey, as bass do not swallow prey larger than their gape width (Hill et al. 2004). After measuring, crayfish were added to streams at 0930. During this timeframe, crayfish spent 14.5 h in mesocosms before any chemical cues were delivered to the systems at 0000. Shelters were then placed in mesocosms. Each shelter was placed approx. 20 cm apart from one another to allow for crayfish to have enough room to explore the mesocosm without being scored as actively using shelters during behavioral video analyses. Next, approximately 5 g (initial weight = 5.01 ± 0.0007 g (mean ± SEM); range of values = 4.99–5.03 g) of *Chara* spp. was dried in a salad spinner and attached to a glass rod (25.5 cm × 0.6 cm (length × outer diameter)) using 26-gauge floral wire. Loaded glass rods were then fixed to cloth brackets (24 cm × 19 cm (length × width)) to prevent loss of *Chara* spp. from water flow before final weight measurements were taken. *Chara* spp. was then placed 7.5 cm away from either the 1- or 4-opening shelter. Water from a 291-*l* bass holding tank (72.4 × 49.8 × 38.6; l × w × h cm) was pumped into a constant head tank in order to expose crayfish to predatory chemical cues from 0000 to 0400. All trials with bass present had holding tanks that housed 2 bass during the experimental period in order to keep chemical cue concentration as similar as possible, as chemical cues enter the water via fish skin, excrement, and gills (Brown et al. 1995; Evans et al. 2005; Glover et al. 2013). Control trials that did not have bass odor present also had water pumped from the 291-*l* tank in order to keep water flow parameters consistent between control and odor-exposed trials. Finally, an automatic light timer activated red lights at 2330 to illuminate mesocosms, and a DVR system began recording behavioral assays each day from 0000 to 0400. This timeframe has been previously shown to be sufficient in capturing the nocturnal behaviors of crayfish (Wood et al. 2018). Waterflow through mesocosms was slow enough that no visible surface distortion was present in recordings.

The next morning, crayfish were removed from streams at 0800. Next, bass were removed from the experimental holding tank and returned to the stock tank. Finally at 0830, *Chara* spp. was removed from mesocosms, dried in a salad spinner, and weighed again to obtain total consumption in each trial. Previous work has shown that any loss of *Chara* spp. due to water flow is negligible in this set up, so change in *Chara* spp. weight can be attributed to consumption (MacKay and Moore 2021; Adami-Sampson et al. 2023). Each experimental holding tank was fully drained and allowed to fill and flush for 1.5 h before new trials began in order to remove odors from previous trials. A total of 12 trials were able to be conducted each day.

### Crayfish behavioral analysis

All videos were analyzed at the end of the experimental period by a single viewer blind to treatment to ensure that consistency of behavioral scoring was maintained throughout the analysis process. Crayfish behavior was then quantified within artificial streams for the total time (s) spent in a shelter (time in shelter), climbing on top of a shelter (time in an area), or foraging. Crayfish were scored as actively participating in these behaviors when at least 50% of the crayfish's carapace was inside of shelters, on top of shelters, or over top of *Chara* spp. In order to map the movement patterns of crayfish, the viewer also recorded information regarding the total number of crayfish transitions from one area to the next (i.e., if the crayfish moved from the 1-opening shelter area to the 4-opening shelter area, this was counted as a transition). Thus, the behavioral analysis produced 7 dependent variables: time in 1- and 4-opening shelters (s), the time in 1- and 4-opening areas (s), total foraging time (s), consumption percent, and the total number of transitions from one area to the next.

### Macrophyte consumption

Consumption of the macrophyte *Chara* spp. was assessed using the following formula:


$$\frac{\text{initial mass-final mass}}{\text{initial mass}}\times 100$$

where the initial mass was determined before trials began, and the final mass was taken on the following day when trials ended. Macrophyte consumption was made into a percentage to account for any possible minor discrepancies in masses throughout the experiment. This process was used across all trials.

### Statistical analysis

Data conditioning and treatment followed the steps outlined by Zuur et al. (2009) for mixed effects modeling. The first step in this process began with the creation of dot charts in order to identify any possible outliers contained within the dataset. Rosner tests were conducted on potential outliers, but removing these values did not significantly alter *P*-values, so no outliers were removed. Next, *q–q* plots, histograms, and Shapiro–Wilk tests were created to represent each variable and examine the underlying distribution. All variables had a non-normal distribution. We used “bestNormalize” to determine the most appropriate transformation to normalize our dataset (Peterson 2021). All dependent variables except for transitions were transformed using OrderNorm transformation, which is a rank-based transformation. Transitions were transformed using Square Root transformation. Time and shelters 1 and 4 and time foraging did not normalize. All other variables became normalized after transforming.

All dependent variables were analyzed using a linear mixed effects model by running the “lmer” function from the “lmerTest” package in R (Kuznetsova et al. 2017; R Core Team 2023). Our analyses produced 2 separate linear mixed effects models. First, for time in shelters and time in areas, models were constructed using 2 categorical factors (fish presence/absence and a treatment factor that combined shelter arrangement with food location) as fixed factors and a nested random effects term. Because fish were placed within bins and this water was pumped into each of 2 stream mesocosms to represent fish presence or absence, bin number was the random effects term in our modeling with mesocosm nested in bin number. The linear mixed models used for time spent foraging, consumption percent, and transitions utilized one categorical and one continuous factor (treatment as mentioned above and average gape ratio) as fixed factors, as well as the same random effects term. We chose to utilize the average gape ratio for 2 reasons. First, previous work has shown that gape ratio is a determinant of perceived risk in bass-crayfish predator–prey relationships (Wood and Moore 2020a; Alexander and Moore 2025). Second, we found that simply including bass presence/absence did not hold any statistical power for these 3 dependent variables, as there were too many pairwise comparisons to show which variables were significant. The fixed effects were evaluated using the anova function from the lmerTest package and the summary function from the base functions of R (Kuznetsova et al. 2017). For significant models, additional post hoc analyses were run using the “emmeans” function with a Tukey adjustment from the “emmeans” package in R in order to determine whether the treatment produced significant effects (Lenth 2023).

## Results

The total time crayfish spent in shelters was significantly impacted by an interaction between the total number of openings on a shelter and the spatial arrangement of both the shelters and *Chara* spp. in mesocosms (*F*_(3,324,0.05)_ = 8.2*, P* < 0.001; Fig. 2). Specifically, regardless of where *Chara* spp. was located, crayfish spent more time in 4-opening shelters when these shelters were upstream (*P* < 0.0001, *β* = −0.63 for upstream *Chara* spp.; *P* = 0.044, *β* = −0.37 for downstream *Chara* spp.). Crayfish also spent significantly more time in upstream 1-opening shelters when *Chara* spp. was also upstream (*P* = 0.02, *β* = −0.42).

**Figure 2:**
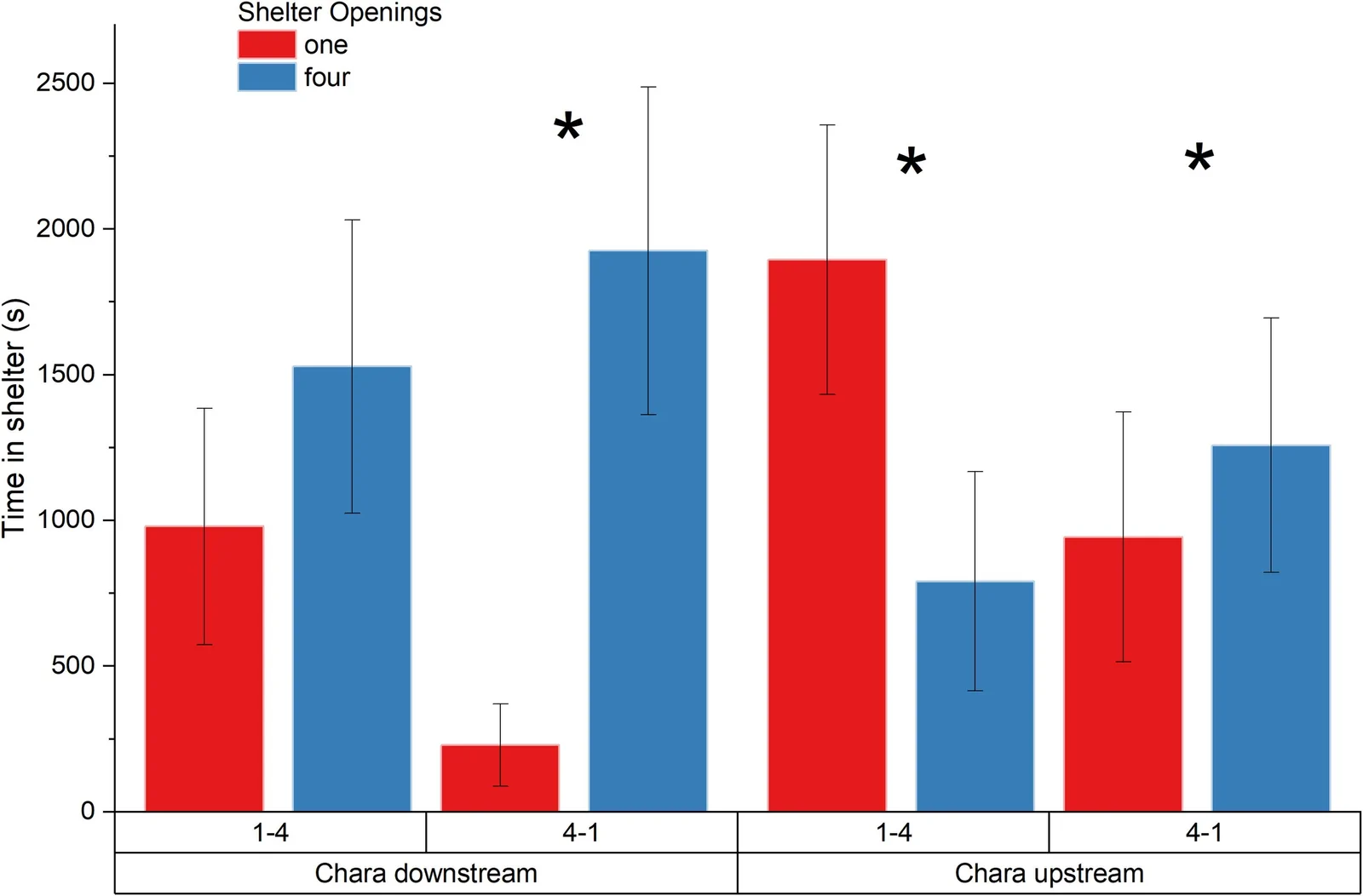
Mean (± SEM) of the total time (s) crayfish spent in each shelter across all spatial arrangements of shelters and *Chara spp*. locations is shown. Solid red bars represent time spent in 1-opening shelters, and blue bars represent time spent in 4-opening shelters. Shelter arrangement is shown as either 1-opening shelter upstream (1-4) or 4-opening shelter upstream (4-1), and *Chara spp*. location is shown as either upstream or downstream next to the shelter Significance is shown by asterisks in each arrangement which was found by first running a linear mixed model (*F*_(3,324,0.05)_ = 8.2) followed by a Tukey post hoc analysis (*P* < 0.05).

Next, we found that time spent in areas around the shelters was significantly affected by an interaction between the total number of openings on a shelter and the spatial arrangement of both the shelters and *Chara* spp. within mesocosms (*F*_(3,319,0.05)_ = 10.5, *P* < 0.001; Fig. 3). Similar to time in shelters, we found that crayfish spent significantly more time in areas near 4-opening shelters regardless of *Chara* spp. location (*P* < 0.0001, *β* = −0.83 for upstream *Chara* spp; *P* = 0.011, *β* = −0.62 for downstream *Chara* spp.). We also found that crayfish spent significantly more time in areas near upstream 1-opening shelters only when *Chara* spp. was also upstream (*P* = 0.0012, *β* = −0.67).

**Figure 3:**
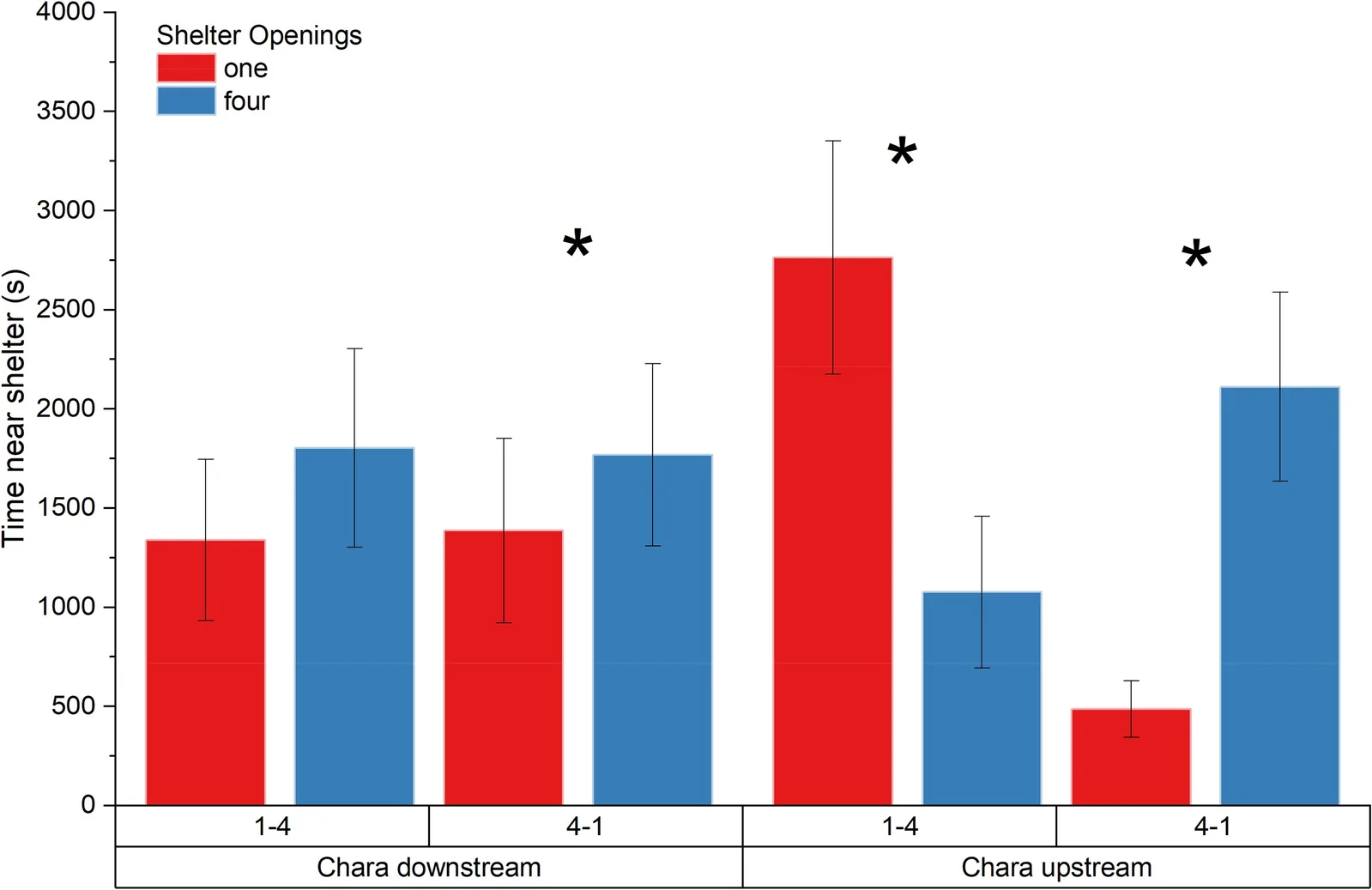
Mean (± SEM) of the total time (s) crayfish spent in areas near shelter type across all spatial arrangements of shelters and *Chara spp*. locations is shown. Solid red bars represent time spent in areas near 1-opening shelters, and blue bars represent time spent in areas near 4-opening shelters. Shelter arrangement is shown as either 1-opening shelter upstream (1-4) or 4-opening shelter upstream (4-1), and *Chara spp*. location is shown as either upstream or downstream. Significance is shown by asterisks in each arrangement which was found by first running a linear mixed model (*F*_(3,319,0.05)_ = 10.5) followed by a Tukey post hoc analysis (*P* < 0.05).

We also found significant affects for the total number of transitions crayfish displayed from one area to the next, the total time crayfish spent foraging, and the amount of *Chara* spp. consumed. First, the number of transitions significantly decreased as the average gape ratio of bass in trials increased (*F*_(1,154,0.05)_ = 6.4, *P* = 0.012, *β* = −0.23; Fig. 4). Next, we found that crayfish total time spent foraging significantly increased as the average gape ratio of bass in trials also increased (*F*_(14,154,0.05)_ = 21.3, *P* < 0.001, and *β* = −0.37; Fig. 5). Finally, the total percent of *Chara* spp. that crayfish consumed in trials significantly decreased as average gape ratio of bass in trials increased (*F*_(1,154,0.05)_ = 11.4, *P* = 0.009, and *β* = −0.38; Fig. 6).

**Figure 4:**
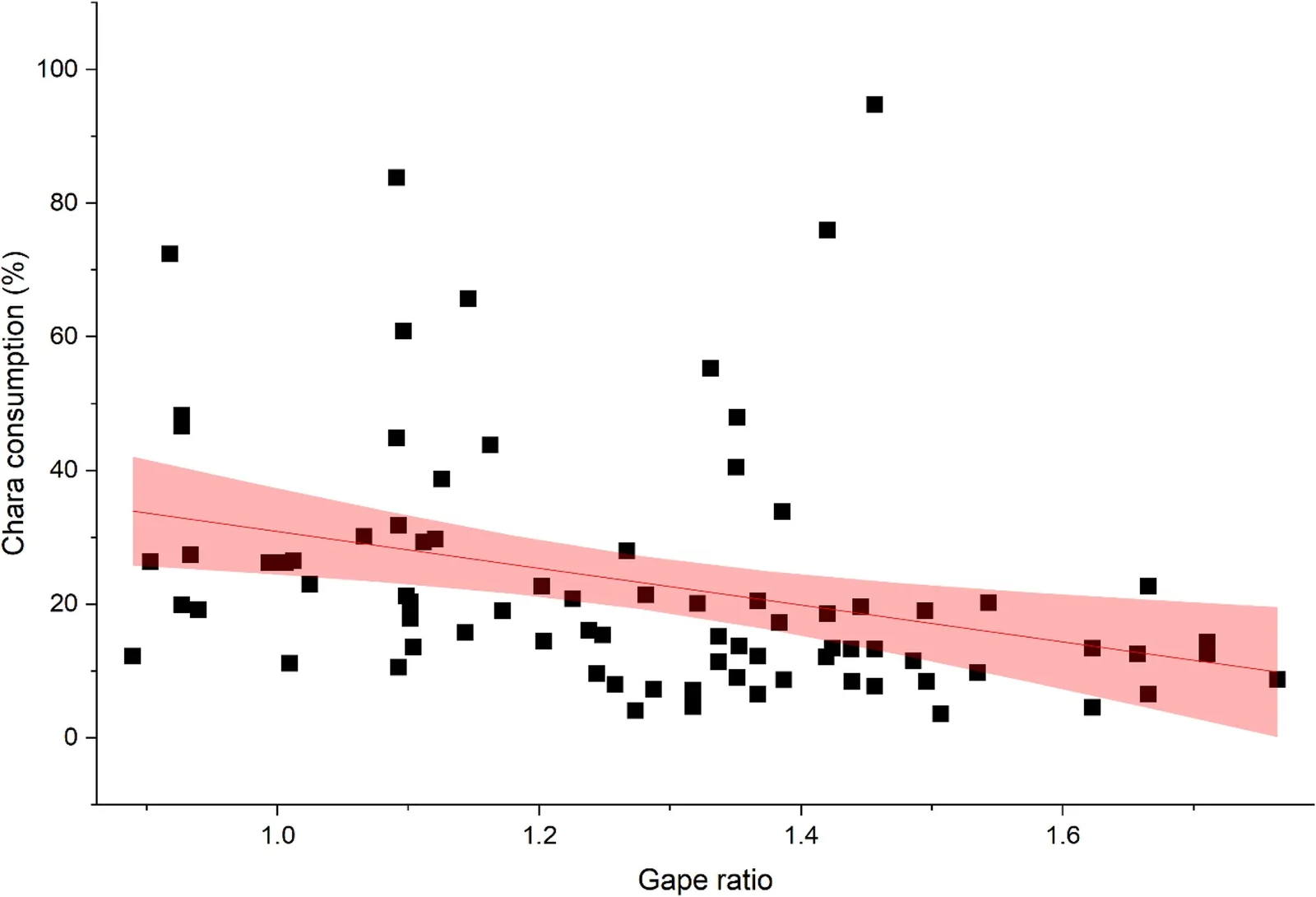
Total transitions crayfish performed from one area to the next as a function of the average gape ratio of bass across trials. Each solid black square is representative of a single trial. The red line and shading represent the best-fit line and the 95% confidence interval. A regression analysis showed *Chara spp*. consumption significantly decreased as gape ratio increased (*r*^2^ = 0.01, *P* = 0.04).

**Figure 5:**
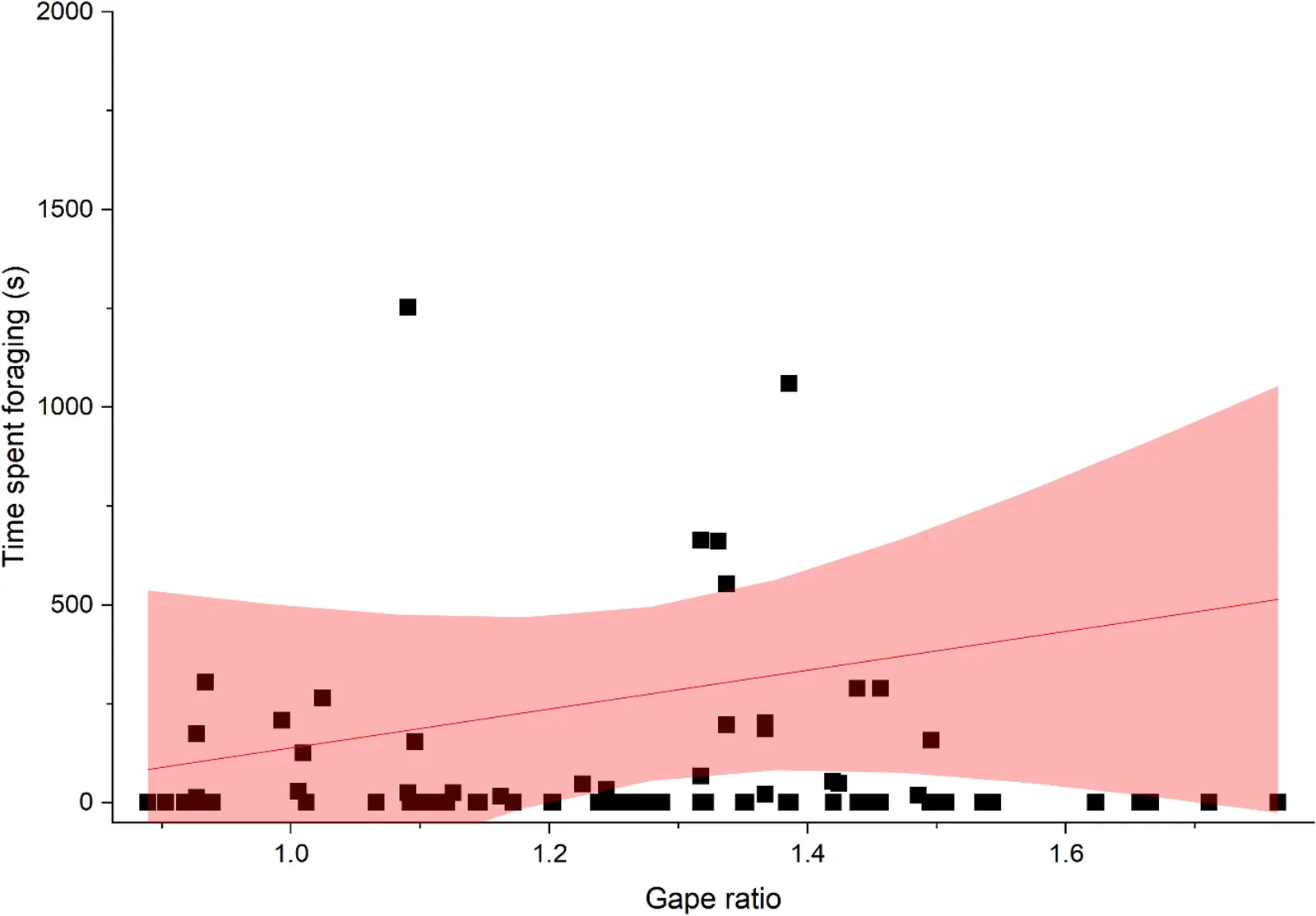
Total time crayfish spent forging as a function of the average gape ratio of bass across trials. Each solid black square is representative of a single trial. The red line and shading represent the best-fit line and 95% confidence interval. A regression analysis showed *Chara spp*. consumption significantly decreased as gape ratio increased (*r*^2^ = 0.06, *P* = 0.04).

**Figure 6:**
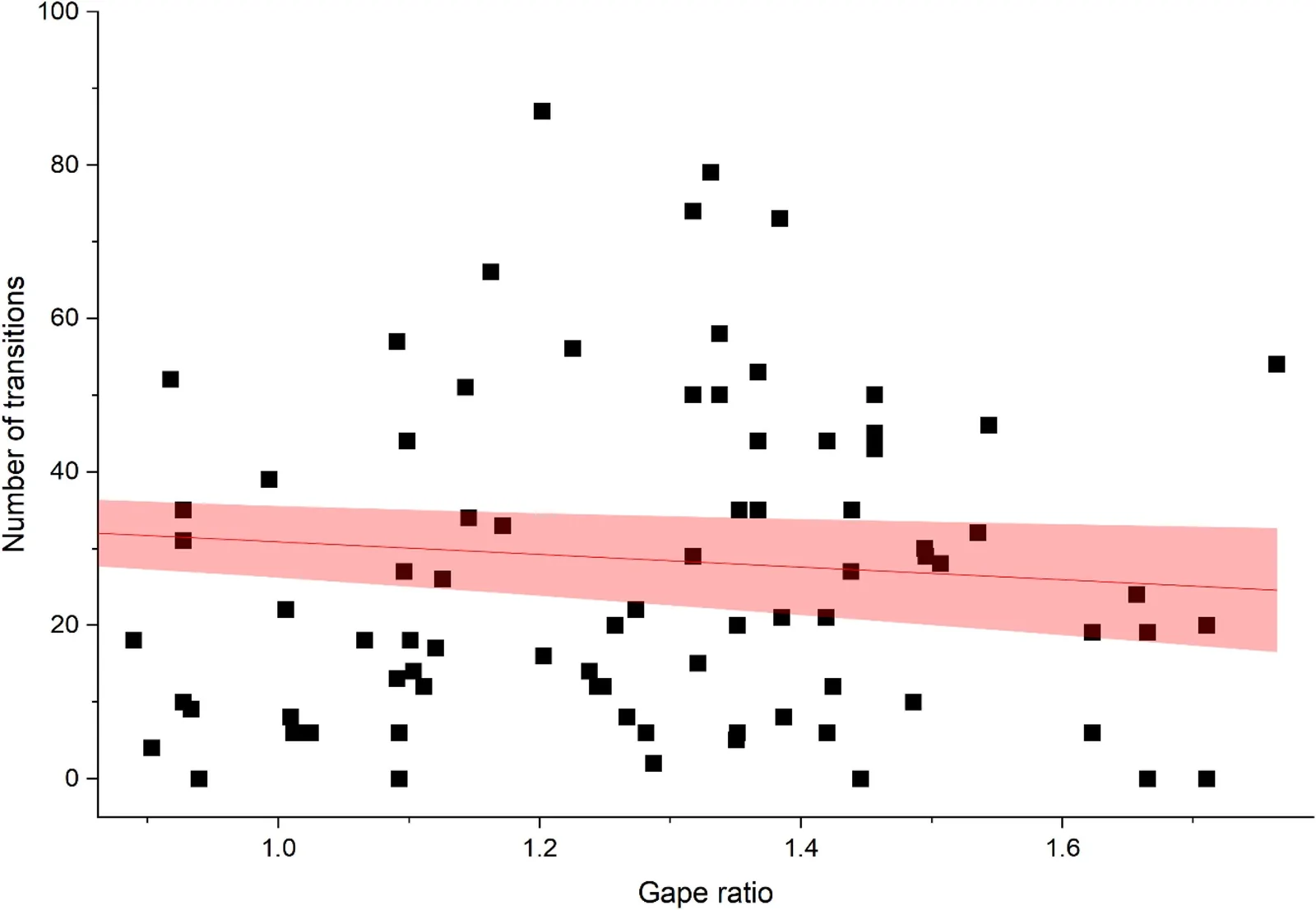
Total percent of *Chara spp*. consumed as a function of the average gape ratio of bass across trials. Each solid black square is representative of a single trial. The red line and shading represent the best-fit line and the 95% confidence interval. A regression analysis showed *Chara spp*. consumption significantly decreased as gape ratio increased (*r*^2^ = 0.09, *P* = 0.004).

## Discussion

We found that crayfish make decisions related to sheltering and foraging based upon food location, shelter quality, and shelter arrangement, but the behavioral suite that follows is dependent upon specific contexts. Though previous work has shown that crayfish prefer shelters with fewer openings (Martin and Moore 2008), we found that crayfish actually utilized 4-opening shelters and the areas around these shelters more often regardless of food location or arrangement of shelters. This may be due to 4-opening shelters having more points of entry/exit for crayfish to utilize. Specifically, crayfish were likely able to move through these shelters much easier than 1-opening shelters, where exiting would include either backing out of the shelter or turning around in the shelter before exiting. Thus, though refuges with fewer openings may technically be “safer”, refuges with more openings may offer increased ease of movement for crayfish. We also found that the only time crayfish used 1-opening shelters more was when these shelters were upstream (nearest cue introduction) and near food. This shows that prey organisms may utilize refuges in high-risk areas but only when those refuges are near a food source and offer a certain level of safety. Our results also showed that the relative risk prey experience directly alters foraging and movement behaviors. For instance, we found that though the overall time crayfish spent foraging increased with increasing risk, the actual amount of food consumed significantly decreased. We also found that crayfish moved from one area of the mesocosm to the next less as risk increased. This information could be indicative of overall decreased movement through the habitat resulting in fewer but lengthier feeding bouts as risk increases. Though this seems counterintuitive, crayfish have been known to increase feeding to grow larger in order to evade capture by gape-limited predators such as bass (Wood and Moore 2020b). Because crayfish used may have been previously exposed to bass in natural setting before being captured, largemouth bass could have potentially been a known predator to some individuals, which may affect behavioral alterations (Kaur et al. 2023). Interestingly, and important for our study, crayfish can determine relative size relationships, number of bass present, and even bass diet simply from chemical cues (Beattie and Moore 2018; Wood and Moore 2020a). Combined together and with the literature, these results show that, although predation risk is certainly a motivator in altering prey behavior, the proximity of food to refuges may also underlie certain behavioral choices.

A basic decision most organisms must make is what ratio of their time budget should be spent searching for food versus sheltering or remaining vigilant. In this way, a tradeoff exists when deciding to participate in a certain behavior (e.g., foraging) and when to seek out a refuge so as not to increase risk. Previous work has shown that certain attributes of a refuge (size, number of openings, location within a habitat, etc.) attract prey organisms to utilize those refuges more often as safety may increase in some way (Christensen and Persson 1993; Persson and Eklov 1995; Bhattacharyya et al. 2015). However, also important to consider is that prey decision-making is based on multiple sources of information and beyond just the characteristics of the predator alone. Prey have been shown to make decisions regarding refuge-use based upon the presence of aversive stimuli (i.e., predator cues) as well as attractive stimuli (i.e., refuges) (Alexander and Moore 2025). In this way, prey combine the perception of risk with the perception of safety during decision-making, but little is known about when exactly a relatively moderate refuge may become more attractive to prey. Typically, we would expect prey to choose more safety if that is the only dimension we include in the prey's thought process. That is, because some refuges offer higher levels of safety from predation, we would assume that prey would more often use these refuges over less safe refuges. However, if a food resource is placed near the less safe refuge, and we include food availability into the decision-making paradigm, the attractiveness of the refuges may be very similar. Information regarding potential resources near refuges may alter prey choice in which refuge to visit and how long prey choose to stay here.

The addition of resources to areas near refuges has been shown to reduce costs associated with refuges and missed foraging opportunities, which, in turn, diminishes some of the negative effects of predation risk (Donelan et al. 2017). Thus, food resources’ proximity to refuges may be important information that prey organisms consider when choosing which refuge to utilize, showing that information from multiple spectrums (e.g., risk, safety, and resource availability) in a habitat is important for prey to consider. When prey miss out on feeding events because risk is high, negative effects related to growth, reproduction, and overall health may be observed (Werner and Anholt 1996; Norrdahl and KorpimÄki 2000; Peacor and Werner 2000). Thus, there is a direct trade-off between the ability to safely consume food and needing to seek out a refuge to avoid predator capture. Our results are indicative that prey organisms may actively choose to utilize refuges nearest to food resources when risk is apparent. We also found that feeding bouts become less frequent but endure for longer time periods in risky scenarios. Taken together, our results show the importance of information regarding the proximity of food resources to refuges in high-risk situations. However, this may only be true for food sources that are fixed and not able to freely move through a habitat. It is important to note that, when an organism's food sources are able to freely move about, the distinction between choosing to be near a refuge or food source and further from a potential predator, may become more convoluted and ensuing behaviors may be species specific. Historically, predator–prey ecology focused solely on the location of a predator in a habitat and the response of prey to the predator's location (Lima and Dill 1990; Peckarsky and McIntosh 1998). Additionally, the idea that the quality of cues may be affected by factors such as size of predator, distance from predator, and even the duration a cue may exist in a system has been found to impact prey behavior (Webster and Weissburg 2009; Van Buskirk et al. 2014). However, more recent work has included the location of refuges into this mix (Klecka and Boukal 2014; MacKay et al. 2021). Information regarding both predators and refuges is undoubtedly important in informing prey on where to move away from and where to move to in a habitat, but the location of food is also an important driver in where prey choose to live. In combining these factors (predators, refuges, and food) into more cohesive parts of a whole as opposed to independent and separate entities, we may be able to better understand and predict the effects predators have on prey populations.

Current interest and modeling techniques in predator–prey ecology involve focusing on characteristics such as Allee effect, cannibalism, fear, and immigration and how prey respond to each of these factors (Diz-Pita and Otero-Espinar 2021). Though this is not an exhaustive list of characteristics that have historically and currently been used to quantify prey behavior (see Cooper et al. 1990; Macdonald et al. 1999; Preisser and Bolnick 2008; Nevai and Van Gorder 2012; Samanta et al. 2016 for more examples), we often use the outcomes of these models to make important decisions on how to conserve predator and/or prey species and the ecosystems in which these organisms live. Certainly, we will never be able to include the infinite number of possibilities of factors that go into altering the interactions between predators and prey. However, including as much relevant information as possible into our thinking and modeling of predator–prey relationships will help us to make better suggestions and predictions about these organisms, as these relationships often exert far-reaching ecosystem effects. For instance, failing to include that prey may not only choose refuges based on physical attributes of the refuge itself, but also the location of refuges in relation to food patches may produce less than optimal conclusions or models based upon specific predator–prey relationships in specific contexts. The results of our experiment show that, in some scenarios, refuge use may be significantly connected to food availability and not just the refuge itself. Thus, this helps in creating a baseline for us to more fully consider the effects that the spatial relationship of refuges and food availability have on prey behavior and predator–prey interactions as a whole.

## Data Availability

Data have been published to the Environmental Data Initiative (EDI) and can be viewed there by readers through this link: https://portal.edirepository.org/nis/mapbrowse?packageid=edi.2027.1.
